# Role of *SALL4* and *Nodal* in the prognosis and tamoxifen resistance of estrogen receptor-positive breast cancer

**DOI:** 10.22099/mbrc.2021.39878.1597

**Published:** 2021-09

**Authors:** Arad Boustan, Fatemeh Mosaffa, Rosa Jahangiri, Hamid Heidarian-Miri, Asefeh Dahmardeh-Ghalehno, Khadijeh Jamialahmadi

**Affiliations:** 1 Department of Medical Biotechnology and Nanotechnology, Faculty of Medicine, Mashhad University of Medical Sciences, Mashhad, Iran; 2 Biotechnology Research Center, Pharmaceutical Technology Institute, Mashhad University of Medical Sciences, Mashhad, Iran; 3 Department of Pharmaceutical Biotechnology, School of Pharmacy, Mashhad University of Medical Sciences, Mashhad, Iran; 4 Department of Epidemiology, School of Health, Mashhad University of Medical Sciences, Mashhad, Iran

**Keywords:** Breast Cancer, Tamoxifen Resistance, SALL4, Nodal, Epithelial to Mesenchymal Transition (EMT), Cancer Stem Cell (CSC)

## Abstract

Despite the discovery of a number of different mechanisms underlying tamoxifen resistance, its molecular pathway is not completely clear. The upregulation of *SALL4* and *Nodal* has been reported in breast cancer. Nevertheless, their role in tamoxifen resistance has not been investigated. In the present study, we compared *Nodal* and *SALL4* expression in 72 tamoxifen sensitive (TAMS) and tamoxifen-resistant (TAMR) patients. Afterward, the correlation of expression data with clinicopathological features and survival of patients was studied. Results showed that both *SALL4* and *Nodal* were significantly upregulated in TAMR compared to TAMS patients. Besides, there was a positive association between *Nodal* and *SALL4* expression. Furthermore, we evaluated their correlation with the expression of *Oct4*, *Nanog* and *Sox2* stemness markers. The results demonstrated that in most tissue samples there was a positive correlation between *Nodal* and *SALL4 *expression with these stemness markers. Besides, the overexpression of *SALL4* and *Nodal* significantly correlated with the N stage. Moreover, the overexpression of *SALL4* was associated with extracapsular invasion and lymphatic invasion. High level expressions of *SALL4* and *Nodal* had a significant association with worse disease-free survival (DFS) rates. In addition, increased level of *Nodal* expression provides a superior predictor factor for DFS. The multivariate Cox regression analysis also revealed that for DFS, perineural invasion (PNI) was independently an unfavorable prognostic value. These findings suggest that the high expression of *SALL4* and *Nodal* could contribute to tamoxifen resistance and worse survival rates in tamoxifen-treated ER^+^ breast cancer patients.

## INTRODUCTION

Breast cancer is the leading cause of cancer-related deaths in the female population worldwide and accounts for 14 percent of all deaths [1]. Similarly, in Iran, breast cancer is the most prevalent cancer in women, responsible for 12.5 percent of all cancer cases. Unlike high-income countries, this malignancy is increasing in the country [2].

The first strategy for breast cancer treatment is local therapy. Usually, patients undergo radiation and breast surgery to eradicate the neoplasm. Moreover, depending on the type of breast cancer, systemic treatments such as chemotherapy, immunotherapy, and hormone therapy might be used. Different endocrine therapy approaches are implemented based on what kind of hormone receptor is expressed on cellular surfaces. For ER^+^ patients, tamoxifen treatment is the preferred choice [3].

Tamoxifen is a safe and the most prosperous anti-tumor agent against breast carcinoma, and it is a member of drugs called estrogen response modulators (SERMs). This substance occupies the binding position of the ER and predominantly acts as an antagonist in breast cancer cells. This phenomenon leads to the elimination of subsequent effects of estrogen on tumor tissues [4]. In addition, tamoxifen could induce expression of TGF-β (as a tumor suppressor agent) and inhibit IGF-I (as a mitogen) in breast cancer patients [5]. Unfortunately, more than 40 percent of patients who receive tamoxifen experience recurrence of the disease. Tamoxifen resistance can be attributed to different mechanisms, the first of which can be the distinct metabolic activation of tamoxifen in different individuals. Consequently, metabolites produced by the tamoxifen metabolism have different binding affinity to ER. Furthermore, the downregulation/loss of ER, restyling in the crosstalk between ER and TGF-β signaling pathway, the imbalance of reactive oxygen species (ROS), specific miRNAs, the ubiquity of cancer stem cells (CSCs), and the epithelial-mesenchymal transition (EMT) are other reported mechanisms involved in tamoxifen resistance. Nevertheless, the potential molecular pathways underlying resistance are still unclear [6, 7]. Therefore, identifying more details of the mechanisms responsible for tamoxifen resistance is crucial for better prediction of clinical outcomes.

EMT is a complex process in which epithelial cells achieve mesenchymal properties. In this process, epithelial cells undergo numerous biochemical alterations such as decreased E-cadherin expression and increased expression levels of Vimentin and N-cadherin, which make cells gain migratory capacity and become more resistant to apoptosis[8]. CSCs are a subpopulation of tumor cells with self-renewing and differentiation ability that have been discovered in different cancers. CSCs are responsible for the progression of a tumor and play a critical role in treatment failure[9]. Since both EMT and CSCs are contributing to drug resistance, tumor recurrence, metastasis, and comparable signaling pathways, it has been suggested that there is a link between CSCs and EMT process [10].

Zinc finger transcriptional factor SALL4 is a member of the spalt-like gene family that is essential for stem cell pluripotency and self-renewal of embryonic stem cells (ESCs). SALL4 is upregulated in various cancers, including gastric cancer, renal carcinoma, leukemia, and breast cancer. *SALL4* can positively regulate stem cell markers *Sox2*, *Oct4*, and *Nanog* to maintain an undifferentiated state. Thus, *SALL4* is responsible for malignancy and recurrence of tumors[11]. *Nanog*, *Sox2*, and *Oct4* are transcriptional factors that prevent differentiation of embryonic stem cells and help them maintain their pluripotency and self-renewal properties. *In-vitro *studies on human and mouse CSCs showed that the downregulation of *SALL4* causes activation of apoptosis cascade [12].

*Nodal* is a member of the highly-conserved TGF-β superfamily, which is crucial during embryogenesis to regulate processes such as cellular organization, left-right axis specification, and particularly the regulation of the mammary gland, which occurs through the activation of mechanisms that involve the CSCs and EMT [13]. Nodal and Nanog signaling pathways have a reciprocal relationship in cellular pluripotency. For instance, Vallier et al. has shown that Nodal signaling repression prevents *Nanog* expression and causes cells to differentiate [14]. Expression of *Nodal* remains only in limited adult tissues such as embryonic tissues and endometrium [15]. The reactivation of *Nodal* expression is associated with tumor recurrence and poor clinical outcomes in several human cancers including ovarian cancer, melanoma, and breast cancer [16].

As described above, both *Nodal* and *SALL4* genes are involved in stemness signaling pathways and might be responsible for the recurrence and resistance of different tumors to treatment. However, studies on the roles of *Nodal* and *SALL4* in tamoxifen resistance are limited and, to our knowledge, there is no published article on the association between the expression of *Nodal* and *SALL4* in ER^+^ breast cancers. The relation of their expression with the clinical outcome in tamoxifen treated breast cancer patients has not been reported. This study has tried to examine the mRNA expression of *Nodal* and *SALL4* in tamoxifen sensitive and tamoxifen-resistant breast carcinomas to discover whether they have any effect on resistance during tamoxifen therapy. Moreover, this research assessed the correlation between the expression of *SALL4* and *Nodal* with the patients’ clinical outcome. 

## MATERIALS AND METHODS

**Ethical Statement:** All procedures performed in this study were approved by the Ethics Committee at Mashhad University of Medical Sciences, Mashhad, Iran, and obtained the ethical code IR.MUMS.MEDICAL.REC.1398.600.

**Tissue Samples: **Complete information for the procedure of sample selection is described in our previous study [17]. Briefly, the patients’ cases that had complete clinicopathological records at Iran’s tumor bank were obtained. Next, ER-positive breast carcinoma patients with tamoxifen as their last stage of treatment were selected. Patients who received hormone therapy or other neoadjuvant therapies before the main treatment were not included in this study. Likewise, ER-negative patients were excluded. The selected patients received tamoxifen for a period of 6 months to 5 years or more. Seventy-two patients were chosen for a retrospective case-control study (Table 1). 

**Table 1:** Clinicopathological characteristics of breast cancer patients

**Features**	**Categories**	**Number of patients**	**Percentage (%)**
Histological grade (MBR)^a^	Grade I Grade II Grade III	25 34 13	34.7 47.2 18.1
T stage	T1 T2 T3 T4	9 44 17 2	12.5 61.1 23.6 2.8
N stage	N0 N1 N2 N3	22 21 18 11	30.5 29.2 25 15.3
Extracapsular *Nodal* extension (ECE)	Yes No	15 57	20.8 79.2
DCIS histology	Comedo type Non-Comedo type	9 63	12.5 87.5
Nipple involvement	Yes No	13 59	18.1 81.9
Lymphatic invasion	Yes No	55 17	76.4 23.6
Perineural invasion	Yes No	30 42	41.7 58.3
ER-status	Positive Negative	72 0	100 0
PR-status	Positive Negative	47 25	65.3 34.7
HER-2 statue	Positive Negative	19 53	26.4 73.6
p53 status	Positive Negative	23 49	31.9 68.1

^a^MBR: Modified Bloom-Richardson

Afterward, these patients were equally divided into two groups: TAMR and TAMS. In the TAMR group, patients showed signs and symptoms of recurrence after 6 months or less during tamoxifen therapy. Symptoms were recurrence of cancer in the breast or opposite breast tissue, metastasis to other tissues including bone, liver, lung, or death. Patients who did not have symptoms of recurrence were included in TAMS. For molecular experiments, fresh tissues were stored at -80°C.

**RNA Extraction and cDNA Synthesis: **The total RNA of breast cancer tissues was isolated using RiboEx^TM^ kit (GeneAll®, Seoul, South Korea) in accordance with the manufacturer's protocol. To realize the purity and concentration of the extracted RNAs, NanoDrop 2000C (Thermo Scientific, USA) was used. The ratio of absorbance at 260/280 and 260/230 of each isolated RNA was approximately 2 and 1.8-2.2, respectively. One microgram RNA was used for cDNA synthesis. With regard to cDNA generation, random hexamer primers were utilized according to the manufacturer's instruction kit (Yekta Tajhiz Azma, Iran). cDNAs were kept at -20 °C until use in RT-qPCR.

**Quantitative Real-Time PCR (QPCR): **RT-qPCR reactions were conducted on the Light cycler®96 system (Roche, Switzerland) by using the SYBR Green master mix (Yekta Tajhiz Azma, Iran). cDNA was amplified by specific sets of primers. To calculate relative gene expression, the 2^-∆∆Ct^ method was used. β-actin (ACTB) was used as the endogenous control gene to normalize background gene expression levels. It was already demonstrated that β-actin is a stable and convenient endogenous control gene in breast cancer tissues and especially is invariant for TAMS and TAMR breast cancer samples [17,18]. Thermal conditions used for amplification comprised an initial denaturation at 95°C for 3 minutes, 40 cycles of amplification: 95°C for 15 seconds, and befitting annealing temperature depending on optimum primer Tm (Table 2) for 30 seconds, 72°C for 1 minute. For confirmation specificity of PCR reaction for each reaction, PCR products were run on a 2% agarose gel. 

**Table 2:** QRT-PCR primer sequences

**Gene**	**Sequences**	**Amplicon length (bp)**	**Tm(°C)**	**Reference**
*Nodal*	Forward: 5'- AGAAGCAGATGTCCAGGGTAGC-3' Reverse: 5'- AGAGGCACCCACATTCTTCC-3'	534	60	[21]
*SALL4*	Forward: 5'- ACCCCGGAGTTTGCAGAT-3' Reverse: 5'- CTTCATCCTCACTCGCCAC-3'	103	58	[22]

**Statistical Analysis: **Statistical analysis was carried out by the Statistical Package Social Science Professional software version 26.0 (SPSS, Chicago, IL, USA) and GraphPad Prism 8.4 software. The Shapiro-Wilk test was conducted for testing data normality. Logistic regression was used to compare and model clinicopathological variables. Gene expression levels between TAMS and TAMR tissues were compared using an independent t-test. The correlation between *Nodal* and *SALL4* were evaluated by Pearson's correlation. Disease-free survival (DFS) and overall survival (OS) rates were assessed by the Kaplan-Meier method. For comparing the differences between the two groups, the Log-rank test was used. For calculating the HR of DFS and OS, the Cox proportional hazard model was used. *P*<0.05 was considered statistically significant.

## RESULTS

mRNA expression of *Nodal* and *SALL4* was evaluated by RT-qPCR. Regardless of tumor type, *Nodal* and *SALL4* were detected in all breast tumor tissues. Values of mean fold change in the mRNA expression of *Nodal* (*P=*0.0009) and *SALL4* (*P=*0.0235) in TAMR patients compared to TAMS patients were 3.01 and 2.82, respectively (Table 3). Moreover, mRNA fold change of individuals was illustrated to depict the exact dispersion of mRNA expression in the population of the study. For indicating different expressions of *Nodal* and *SALL4* between two groups, a scatter plot of ∆Ct amounts was drawn (Fig. 2). It unarguably demonstrated that there is an upregulation of *Nodal* and *SALL4* in TAMR patients. 

**Table 3:** Mean fold increase of expression levels of Nodal and SALL4 in TAMR tumor tissues (N=36) compared to tamoxifen sensitive tissues (fold induction was normalized to β-actin)

**Gene**		**TAMR**	**TAMS**	**Fold change**
Nodal	Mean of ∆Ct ±SD	3.29±1.42	4.88±2.1	3.01
SALL4	Mean of ∆Ct ±SD	13.65±2.73	15.12±2.65	2.82

**Figure 1:**
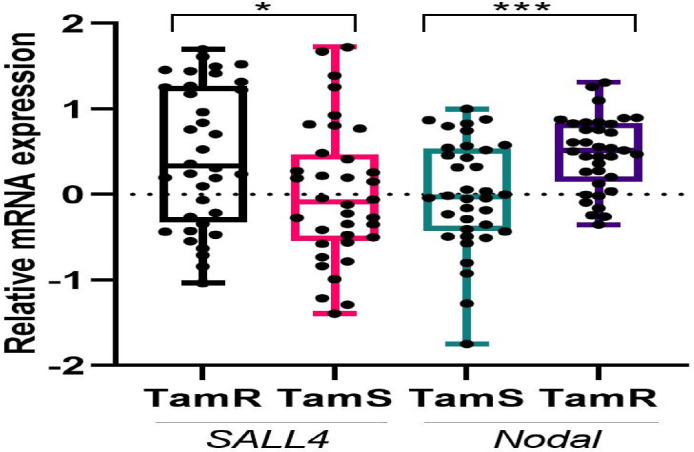
Student t-test was performed to analyze the mRNA expression of *Nodal* and *SALL4* between TAMR and TAMS in the population. The mRNA expression was evaluated by qRT-PCR and normalized to β-actin. ***p<0.001, *p<0.05

**Figure 2:**
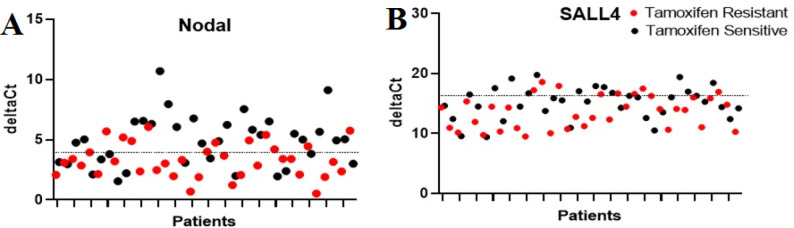
The scatter plot of delta-CT values of Nodal(a) for SALL4(b) and TAMS and TAMR patients and controls. Higher delta-CT value represents lower expression of the gene at the mRNA level

**Figure 3:**
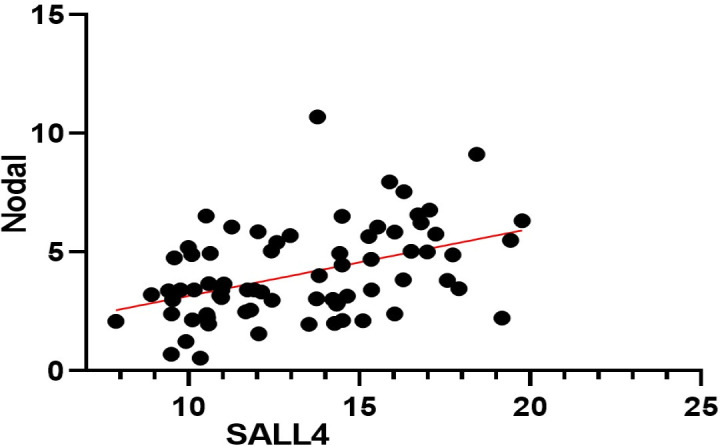
Regression plot presenting a positive correlation between Nodal and SALL4 (P=0.001, r= 0.4336). X and Y-axis illustrates relative expression normalized to β-actin

Data obtained from the Spearman correlation coefficient showed that there was a significant correlation between *Nodal* and *SALL4* mRNA expression (r=0.4336, *P*=0.001). This significant correlation was illustrated as a scatter plot (Fig. 3).

In our previous publication, we had determined the expression of *Nanog*, *Sox2*, and *Oct4* in the same population at the mRNA level [17]. Hence, the correlation analysis between *SALL4* and *Nodal* expression with mentioned genes were assessed. These results demonstrated that *Nodal* expression associates with two stemness markers including *Nanog* (*P=*0.018) and *Sox2* (*P=*0.001). Moreover, the mRNA expression of *SALL4* also significantly correlates with *Nanog* (*P=*0.038) and *Sox2* (*P=*0.001). We could not find any conclusive result that explains *Oct4* correlates with *SALL4* or *Nodal *[23].

We evaluated the relationship between the expression of *SALL4* and *Nodal* with the clinicopathological features of breast cancer patients by logistic regression. The statistical analysis revealed that both *SALL4* and *Nodal* upregulation significantly associate with the N stage (*P=*0.016 and *P=*0.001). Besides, the results have demonstrated that *SALL4* correlates with extracapsular invasion (*P=*0.052) and lymphatic invasion (*P=*0.038). However, there was no association of *SALL4* and *Nodal* expressions with other clinicopathological features (Table 4).

**Table 4:** Correlation between expression levels of SALL4 and Nodal with clinicopathological features

**Patient feature**	***SALL4***								***Nodal***		
	**High**	**Low**	**95% CI**	**OR**	***P***		**High**	**Low**	**95% CI**	**OR**	***P***
Grade Grade 1 Grade 2,3	13(32%) 28(68%)	14(45%) 17(55%)	0.61-4.22	1.61	0.329		13(48%) 27(52%)	14(43%) 18(57%)	0.61-4.22	1.61	0.329
N stage N0 & N1 N2 & N3	20(48%) 21(52%)	24(78%) 7(22%)	1.27-10.1	1.55	0.016*		17(43%) 23(57%)	27(84%) 5(16%)	2.33-22.8	7.30	0.001*
T stage T1 & T2 T3 & T4	30(73%) 11(27%)	23(65%) 8(35%)	0.36-3.04	1.05	0.922		29(73%) 11(27%)	24(57%) 8(43%)	0.39-3.28	1.13	0.81
Extracapsular nodal extension Yes No	12(29.2%) 29(70.8%)	3(9.6%) 28(90.4%)	0.66-1.01	0.25	0.052		11(27%) 29(73%)	4(10%) 28(90%)	0.10-1.32	0.37	0.128
DCIS histology Comedo type Non comedo	13(31.7%) 28(68.3%)	13(41.94%) 18(58.06%)	0.58-4.10	1.55	4.105		11(27%) 29(73%)	15(46%) 17(53%)	0.87-6.20	2.32	0.92
Nipple involvement No Yes	35(85%) 6(15%)	25(80%) 5(20%)	0.40-4.85	4.85	0.596		31(77%) 9(33%)	29(90%) 3(10%)	0.08-1.44	0.35	0.149
Lymphatic invasion No Yes	6(15%) 35(85%)	11(35%) 20(65%)	0.10-0.97	0.31	0.044*		10(25%) 30(75%)	7(22%) 25(78%)	0.39-3.58	1.19	0.757
Perineural invasion (PNI) No Yes	24(59%) 17(41%)	18(58.06%) 13(41.94%)	0.39-2.62	1.02	0.96		21(53%) 19(47%)	21(65%) 11(35%)	0.22-1.50	0.57	0.263
PR status Positive Negative	28(61%) 13(39%)	19(61%) 12(39%	0.51-3.61	1.36	0.35		25(63%) 15(37%)	22(68%) 10(32%)	0.28-2.02	0.75	0.58
HER-2 status Positive Negative	9(22%) 32(78%)	10(48%) 21(52%)	0.20-1.69	0.59	0.328		13(33%) 27(67%)	6(23%) 26(81%)	0.69-6.31	2.08	0.193
P53 status Positive Negative	10(24%) 31(76%)	13(42%) 18(58%)	0.16-1.22	0.44	0.117		13(33%) 27(67%)	10(31%) 22(69%)	0.39-2.87	1.05	0.910

* Statistically Significant

**Figure 4:**
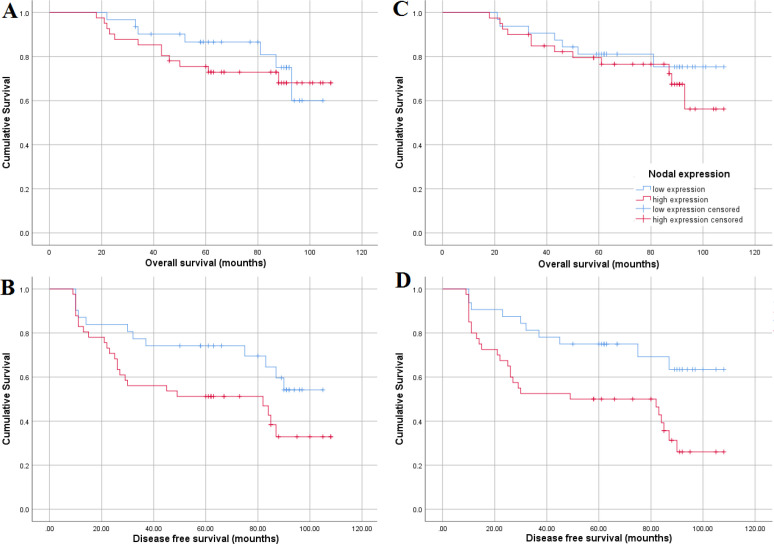
The Kaplan-Meier (KM) survival curves for OS and DFS breast cancer patients in relation to SALL4 (a & b) and Nodal (c & d) mRNA levels

For the investigation of survival analysis, the patients were divided into two groups based on the mRNA amounts of *SALL4*: High mRNA expression versus low mRNA expression. Next, the prognostic values of *SALL4* were evaluated by Kaplan-Meier plots. Kaplan-Meier curves (Figure 4 a-b) illustrated that *SALL4* expression was associated with DFS (*P=*0.049) but not with OS (*P=*0.535). Similar to the analysis of *SALL4*, the patients were first divided into two groups of low expression of *Nodal* versus high expression of *Nodal*. Data from Kaplan-Meier plots (Figure 4 b-c) showed a significant correlation between the expression of *Nodal* and DFS (*P=*0.006). However, *Nodal* was not associated with OS (*P=*0.433).

To determine the dependent association between *Nodal* and *SALL4* mRNA expressions on survival in tamoxifen-treated patients, the Cox proportional hazard regression was performed. The outcome demonstrated that the overexpression of *Nodal* could be a commendatory predictor factor for DFS (HR=2.676, 95% CI:1.287-5.556; *P=*0.008). However, *Nodal* was not associated with OS prognosis. Results from the Cox regression of *SALL4* expression showed that *SALL4* was not significantly correlated to OS (HR=0.746 95% CI=0.294-1.895; *P=*0.538) and DFS (HR=0.505 95% CI=0.251-1.017; *P=*0.505, Table 5). Subsequently, we included significant features of univariate Cox regression including N stage, perineural invasion (PNI), extracapsular *Nodal* extension (ECE), and *Nodal *expression in the multivariate Cox regression model. Data from the multivariate Cox regression showed that for DFS, PNI (HR:0.488;95% CI:0.249-0.954; *P=*0.036) still endures as an unfavorable prognostic factor.

## DISCUSSION

Despite the effectiveness of tamoxifen in the treatment and prevention of ER^+ ^breast cancer patients, more than 40 percent of patients experience disease recurrence [6]. One of the main reasons for poor prognosis and therapeutic resistance is the interrelationship between EMT and CSCs formation. *SALL4* and *Nodal* are both involved in tumorigenesis, progression, drug resistance, and aggressiveness of some human tumors by maintaining CSCs and EMT properties. In this study, we tried to investigate the impact of the expression of *SALL4* and *Nodal* on clinicopathological features and clinical outcome in ER^+^ tamoxifen-treated breast cancer patients [21, 22]. 

*SALL4* promotes EMT by positive regulation of *ZEB1*, *Slug*, *Snail*, and *Vimentin* [22]. Interestingly, SALL*4* associates with *Nanog*, *OCT4*, and *Sox2* [23, 24] to enable breast cancer cells to acquire stem‐cell‐like and metastatic properties. In line with this finding, we showed an association between *SALL4* expression with *Nanog* and *Sox2* expressions. Silencing of *SALL4* in lung cancer and MCF-7 breast cancer cells, increased their sensitivity toward anti-cancer drugs [13, 25]. Furthermore, the period until disease recurrence was shorter in the patients with overexpressed SALL4. These results are consistent with the findings of current research that *SALL4* mRNA was significantly increased in TAMR patients compared to TAMS patients.

**Table 5:** Univariate Cox regression models for DFS and OS in ER^+ ^tamoxifen-treated breast carcinoma patients

Factor of base model	Univariate Cox regression model for DFS HR 99% CI P-value				Univariate Cox regression model for OS HR 99% CI P-value		
Histological Grade (MBR) Grade I Grade II Grade III	2.03 2.01	0.91-4.52 0.77-5.22	0.081 0.149		3.96 3.20	1.10-14.2 0.71-14.3	0.035 0.128
T stage T1 T2 T3 T4	0.69 1.19 1.43	0.25-1.85 0.41-3.43 0.16-12.3	0.464 0.747 0.741		0.52 1.00 2.48	0.14-1.95 0.24-4.03 0.25-24.0	0.338 0.999 0.433
N stage N0, N1 N2, N3	2.32	1.2-4.50	0.012		1.85	0.75-4.57	0.182
Extracapsular *Nodal*							
extension Absent Present	2.17	1.05-4.46	0.035		2.00	0.76-5.27	0.160
DCIS histology Non-Comedo type Comedo type	1.01	0.39-2.62	0.972		1.84	0.60-5.55	0.280
Nipple involvement Absent Present	1.41	0.58-3.43	0.44		1.09	0.31-3.81	0.881
Lymphatic invasion Absent Present	1.78	0.74-4.29	0.196		1.82	0.53-6.26	0.339
Perineural invasion (PNI) Absent Present	2.32	1.19–4.49	0.013		0.78	0.31-1.99	0.614
PR-status Positive Negative	1.15	0.58-2.28	0.677		2.46	0.99-6.10	0.051
HER-2 status Positive Negative	1.14	0.51-2.52	0.739		1.22	0.40-3.70	0.723
P53 status Positive Negative	0.57	0.26-1.21	0.145		1.00	0.38-2.64	0.994
*Nodal* Expression	2.67	1.28-5.55	0.008		0.43	0.57-3.68	0.437
*SALL4* Expression	0.50	0.25-1.01	0.056		0.74	0.29-1.89	0.538

The *SALL4* level in metastatic lymph nodes pertinent to the primary site is a considerable survival prognosis marker in breast cancer [22]. Yue et al., in the assessment of 160 invasive ductal carcinoma patients, demonstrated that *SALL4* expression was associated with lymph node metastasis, ER, PR, HER2, and tumor invasion. Their research showed that *SALL4* was correlated with worse overall survival [26]. In agreement with the previous studies, our results showed that the expression of *SALL4* was associated with lymphatic invasion and N stage. However, we could not find a significant association between *SALL4* expression and ER, PR, and HER2 status. Furthermore, KM analysis demonstrated that the overexpression of *SALL4* was related to worse DFS. 

Nodal protein is an essential embryonic morphogen that is a member of the TGF-β superfamily. During embryonic development, expression of *Nodal* is critical for maintenance of pluripotency of ESCs and effectively increases cell migration through EMT [27]. *Nodal* is silenced in most human organs and is only expressed in restricted to reproductive tissues and ESCs. *Nanog* and *Oct4* promote *Nodal* signaling pathway [27, 28]. Strizzi et al. reported that blocking *Nodal* signaling by knocking down *Nodal* in human triple-negative breast cancer cells (MDA-MB-231) reduced invasive tumor cells and led to apoptosis [29]. Besides, it has been shown that *Nodal* and its receptor are present in prostate epithelial stem cells and prostate cancer cells, and they may have autocrine and paracrine effects on migratory properties and cell proliferation in different tumor stages [30]. In another study, *Nodal* was overexpressed in human colon cancer tissues compared to adjacent normal colon tissues [31]. These results are in line with our results that showed the *Nodal* expression was detected in all breast tumor tissues and its expression was higher in TAMR patients. In a study on breast cancer patients, Strizzi et al. indicated that the *Nodal* expression was remarkably higher in malignant breast samples than benign breast tissues. They showed that *Nodal* expression significantly correlates with higher grades, advanced stage, and lymph node metastasis. However, they could not find a significant association between *Nodal* expression and levels of ER or PR [32]. Interestingly, our data indicated that *Nodal* expression was only associated with the N stage and similar to the previous finding, neither ER nor PR expression had a significant correlation with the expression of *Nodal*. KM survival analysis demonstrated that overexpression of *Nodal* in TAM-treated patients correlates with a worse prognosis in DFS. The analysis of Cox regression showed that *Nodal* expression could independently have a predictive value.

*Nodal* and *SALL4* were simultaneously upregulated in TAMR patients, and statistical analysis indicated that overexpression of *SALL4* and *Nodal* had a significant correlation with each other. On the one hand, both genes play a fundamental role in the progression of EMT and CSCs properties, and previous studies have shown that they have an association to other stemness genes such as *Oct4, Nanog,* and *Sox2*. The researchers surmise that a correlation might exist between *Nodal* and *SALL4* signaling pathway in TAMR patients.

To conclude, it can be asserted that the overexpression of *Nodal* and *SALL4* was associated with a poor prognosis in DFS. Likewise, for the first time, the research illustrated that the expression of *Nodal* and *SALL4* plays a key role in TAMR tumors. According to these observations, a positive correlation existed between *SALL4* and *Nodal* expressions with *Sox2* and *Nanog* expressions. The hypothesis stated that *SALL4* and *Nodal* should have a direct correlation with stemness factors, including *Sox2* and *Nanog*. However, further investigation is needed better to understand the mechanism of *SALL4* and *Nodal* co-expression.
